# Recommendations for post-surgical thyroid ablation in differentiated thyroid cancer: a 2015 position statement of the Italian Society of Endocrinology

**DOI:** 10.1007/s40618-015-0375-7

**Published:** 2015-08-12

**Authors:** F. Pacini, E. Brianzoni, C. Durante, R. Elisei, M. Ferdeghini, L. Fugazzola, S. Mariotti, G. Pellegriti

**Affiliations:** Department of Medical, Surgical and Neurological Sciences, University of Siena, Siena, Italy; Nuclear Medicine Unit, Ospedale Civile di Macerata, Macerata, Italy; Department of Internal Medicine and Medical Specialties, University of Rome Sapienza, Rome, Italy; Endocrine Unit, Department of Clinical and Experimental Medicine, University of Pisa, Pisa, Italy; Nuclear Medicine Unit, University of Verona, Verona, Italy; Endocrine Unit, Fondazione IRCCS Ca’ Granda Policlinico, Milan, Italy; Department of Pathophysiology and Transplantation, University of Milan, Milan, Italy; Department Medical Sciences “M. Aresu”, University of Cagliari, Cagliari, Italy; Endocrinology Division, Garibaldi Nesima Hospital, Palermo, Italy; Department of Endocrinology, University of Siena, Via Bracci, 53100 Siena, Italy

**Keywords:** Radioiodine, Thyroid ablation, Thyroid cancer, Thyroglobulin, rhTSH

## Abstract

**Abstract:**

Post-surgical ablation of thyroid remnant with radioactive iodine (RAI) in differentiated thyroid cancer (DTC) is aimed to destroy any thyroid remnant in the thyroid bed (*remnant ablation*) and any microscopic foci of cancer cells eventually present within the thyroid remnant (*adjuvant therapy*). The present text is an attempt to offer practice guidelines for the indication of thyroid ablation and the preparation of DTC patients considering the latest achievement in the field and the changing epidemiology of DTC observed in the last 10 years.

**Methodology:**

The executive committee of the Italian Society of Endocrinology appointed a task force of thyroid cancer expert including Nuclear Medicine Physicians and Endocrinologists to provide a consensus on the post-surgical ablation in thyroid cancer patients. The task force had no conflict of interest and had no commercial support. A number of specific topics were selected and the members selected relevant papers by searching in the Pubmed for articles published from 2000 to January 2015. Selected studies were categorized by level of evidence, and the recommendations were graded according to the level of evidence as high (A), moderate (B), or low (C).

## Introduction

Post-surgical ablation of thyroid remnant with radioactive iodine (RAI) (*remnant ablation*) in differentiated thyroid cancer (DTC) is aimed to facilitate the early detection of recurrence based on serum thyroglobulin (Tg) measurement and/or RAI whole-body scan (WBS) and to obtain a post-therapy WBS, whose results may change the initial staging by identifying previously undiagnosed disease. In addition, RAI ablation may represent an *adjuvant therapy* by cleaning persistent microscopic foci of cancer which can be present in the thyroid remnant especially in papillary thyroid cancer (PTC) that are frequently multifocal. While the first aim, remnant ablation, is related to follow-up in any patient regardless of his specific risk, the second one, adjuvant therapy, is advocated as a tool to reduce the rates of disease recurrence or cause-specific mortality [1], and thus its use must be justified according to a real risk of recurrence. Indeed, in patients at the lowest risk for recurrence and mortality several studies have shown no benefit of remnant ablation [2]. Benefit becomes evident in patients considered at intermediate [3] or high risk, particularly in terms of reduced recurrence rate and possibly of reduced mortality.

Based on this consideration, several authoritative guidelines have restricted the indication for RAI ablation to specific categories of patients considered at significant risk of recurrence or death. Since selection of patients for thyroid ablation is not the aim of this guideline, we refer to them for further reading.

Here we will limit our discussion to the choice of the most appropriate activity of ^131^I (low or high) to be used whenever thyroid ablation has been decided, including the definition of successful thyroid ablation and the method of preparation for RAI administration.

### Definition of successful remnant ablation

In the past, successful remnant ablation was defined as the absence of visible RAI uptake on a diagnostic RAI WBS performed 6–12 months after remnant ablation. With the introduction of serum Tg measurement and neck ultrasound in clinical practice, the use of performing diagnostic WBS has been largely abandoned. Indeed, both serum Tg determination [4–8] and neck sonography examination [9] are much more sensitive compared to diagnostic WBS in detecting the existence of persistent or recurrent disease [4–8]. Nowadays, the most accepted definition of successful thyroid ablation is an undetectable stimulated serum Tg (or an undetectable basal serum Tg if using ultra-sensitive Tg assays) combined with undetectable serum anti-thyroglobulin antibodies (TgAb) and a negative neck ultrasound.

TgAb are commonly identified in patients with differentiated follicular cell-derived thyroid cancer. Their frequency in patients with DTC is approximately 20–25 % [10]. In case of positive TgAb, serum Tg cannot be use as a predictor of ablation and we have to rely on neck ultrasound and on the trend of serum TgAb. A diagnostic WBS may be considered in this setting. Antibody levels may serve as a surrogate biochemical marker of disease persistence and response to therapy. However, the timing of testing and the duration to see a maximal response appear to differ from Tg levels in patients without TgAb. There may be an initial transient rise in TgAb titer after radioactive iodine (RAI) treatment [11]. Also, it has been shown that the eventual disappearance of TgAb takes approximately 2–3 years on average. Complete ablation of thyroid tissue with its antigenic components results in the disappearance of antibodies to all major thyroid antigens [10–12].

### Preparation for RAI ablation

Remnant ablation has been traditionally performed after thyroid hormone withdrawal to increase endogenous thyroid-stimulating hormone (TSH) to levels sufficient to induce robust RAI uptake in thyroid cells. Empirically, it is estimated that a TSH of >30 mU/L is a good cut off [13], but no comparative study has ever been done to document this assumption.

For thyroid hormone withdrawal, two possible approaches are used: switch from levothyroxine (LT_4_) to triiodothyronine (LT_3_) for some weeks [5, 6] and then stop LT_3_ for 2 weeks, or stop LT_4_ for 3–4 weeks without switching to LT_3_. These two methods have not been compared in terms of better outcomes of ablation.

Since several years, the alternative way of preparation for RAI ablation is the administration of exogenous recombinant human TSH (rhTSH), after a prospective, multicenter, randomized study demonstrated that ^131^I remnant ablation with 100 mCi was equally effective after rhTSH stimulation or thyroid hormone withdrawal [14]. In another study, ablation rates were similar with either withdrawal or preparation with rhTSH using 50 mCi of ^131^I [15]. Additional evidence that preparation with thyroid hormone withdrawal or rhTSH has the same ablation outcome using both high or low radioiodine doses has been provided in two prospective randomized multicenter studies, one in France and one in the UK [16, 17]. In addition, short-term recurrence rates have been found to be similar in patients prepared with thyroid hormone withdrawal or rhTSH [18, 19]. The preparation with rhTSH significantly improves quality of life [14, 20], and reduces both whole-body irradiation [21, 22] and hospitalization time [23]. A recent metanalysis confirms the above results [24]. Nowadays, the use of rhTSH is approved for remnant ablation, with any 131I dose, both in the United States and Europe.

## Recommendation 1

Patients undergoing RAI ablation should be preferentially prepared by rhTSH administration. Thyroid hormone withdrawal may be considered whenever rhTSH is not available or not affordable. Recommendation rating: A.

### Which is the best activity of ^131^I to be employed for post-surgical thyroid remnant ablation

Although there is a trend toward higher ablation rates with higher activities [25, 26], activities between 30 and 100 mCi of ^131^I generally show similar rates of successful remnant ablation [27–30]. A randomized study using preparation with rhTSH showed that ablation rates were comparable with 50 mCi compared to 100 mCi [31]. A prospective, randomized study performed in 160 patients, comparing ablation with 30 and 100 mCi, after preparation with thyroid hormone withdrawal, found no difference in both the ablation rate and in the rate or recurrence during follow-up [32]. Recently, two prospective randomized studies in very large number of patients conducted in France and in the United Kingdom found no significant difference in the remnant ablation rate using 30 or 100 mCi of ^131^I, either after preparation with thyroid hormone withdrawal or rhTSH [16, 17]. It is worth noting, that these two studies included not only low-risk patients, but also patients at intermediate risk of recurrence, including those showing minimal extrathyroidal extension of the primary tumor [17] or lymph node metastases [16, 17]. Also in this category the authors found no difference between low- and high-RAI doses in terms of ablation success rates. This finding has been confirmed in a retrospective study including only patients at intermediate risk, treated with low- or high-RAI activities [33]. In this study, the authors were also able to demonstrate that, with regard to recurrences or deaths, the long-term outcome was not affected by the ablation dose (low or high). Concerning the issue of the follow-up of patients treated with low activity of 131-I and rhTSH or LT4 withdrawal, a recent study [19] showed that in 10 years of follow-up, the rate of recurrence was as low as expected (3.5 %) and similar in both groups. Moreover, the final outcome of these patients was similar at the end of follow-up (Table 1).

**Table 1:** Guidelines for radioiodine thyroid ablation

	Recommendation	Rating
Preparation for RAI ablation	1. Patients undergoing RAI ablation should be preferentially prepared by rhTSH administration [14, 16, 17]	A
Which is the best activity of ^131^I to be employed for post-surgical thyroid remnant ablation	2. The minimum activity (30 mCi) necessary to achieve successful remnant ablation should be utilized, particularly in patients at low risk and intermediate risk [31–33]	B
	3. In patients considered at high risk for recurrence, or if residual microscopic disease is suspected or documented, higher activities (100 mCi or more) should be considered [40]	C
Diagnostic RAI scanning before ablation	4. Pre-ablation diagnostic scans are seldom informative as far as the decision to ablate is concerned. They may be considered when a sustained suspicion of local or distant metastases is present to better define the activity of RAI to be administered or in patients with elevated levels of serum TgAb [38, 40–42]	C
Post-operative serum Tg levels and neck US	5. Post-operative serum Tg levels (in the absence of serum TgAb) and neck US may give additional information regarding the need for ablation and the radioiodine dose to be administered [47, 48]	C
Is a low-iodine diet necessary before remnant ablation?	6. At least in countries with mild or moderate iodine deficiency, there is no need to prescribe a low-iodine diet before remnant ablation. Avoidance of iodine-containing drugs or contrast agents is mandatory [53]	B
Post-therapy WBS after remnant ablation	7. A post-therapy WBS is recommended following remnant ablation. This is typically done 3–7 days after the therapeutic dose is administered [54, 55]	A

No definite studies are available in pediatric patients. For every age patients, some authors have suggested to use a lesion dosimetry or to give the highest dose administrable based on the radiation exposure to the critical organs at risk, especially in high-risk group; others suggest empiric dosage and others an activity based on the patient’s body weight [34–37].

## Recommendation 2

The minimum activity (30 mCi) necessary to achieve successful remnant ablation should be utilized, particularly in patients at low risk and intermediate risk. Recommendation rating: B.

## Recommendation 3

In patients considered at high risk for recurrence, or if residual microscopic disease is suspected or documented, higher activities (100 mCi or more) should be considered. Recommendation rating: C.

In such patients at high risk, some authors have advocated the use of 124I PET as a better diagnostic procedure compared to 131I whole-body scanning in staging disease burden. Relative to 131I planar whole-body imaging, 124I PET identified as many as 50 % more foci of radioiodine uptake suggestive of additional residual thyroid tissue and/or metastases in as many as 32 % more patients [38]. Thus, when available, a 124I PET/CT could be used to perform dosimetry, to tailor treatment, instead of using fixed activities, and to evaluate mean absorbed doses both to target lesions and to non-target organs (salivary glands).

### Should a diagnostic RAI scanning be performed before ablation? Should post-surgical serum Tg levels be considered in decision making?

A diagnostic RAI WBS provides information on the presence of iodine-avid thyroid tissue, which may represent the normal thyroid remnant or the presence of residual disease. There is an increasing trend to avoid diagnostic RAI scans because of its low impact on the decision to ablate, and because of concerns over ^131^I-induced stunning of normal thyroid remnants [39] and distant metastases from thyroid cancer [40–42]. The alternative radiopharmaceutical for staging, 123I, is not readily available and has a short half-life. [43–45].

## Recommendation 4

Pre-ablation diagnostic scans are seldom informative as far as the decision to ablate is concerned. They may be considered when a sustained suspicion of local or distant metastases is present to better define the activity of RAI to be administered or in patients with elevated levels of serum TgAb. In any case, diagnostic scans should be performed using tracer doses of ^123^I (if available) or low ^131^I activity (1–2 mCi). In the last case, the therapeutic activity should be delivered within 72 h of the diagnostic activity to avoid stunning. Recommendation Rating: C.

### Post-operative serum Tg levels and neck US

Some studies investigating the clinical significance of post-operative serum Tg levels have shown that low post-operative levels of non-stimulated Tg does not exclude persistent disease [46]. However, serum Tg levels may be useful for the indication of 131I activity to be delivered [47] and may represent a significant prognostic marker [48, 49].

Similar prospective information may be derived from the execution of neck US before ablation. US should be performed to check the central and lateral compartments of the neck whenever a cancer is fortuitously discovered at histology, or a preoperative US has not been done or in the presence of high pre-ablation serum Tg values.

## Recommendation 5

Post-operative serum Tg levels (in the absence of serum TgAb), and neck US may give additional information regarding the need for ablation and the radioiodine dose to be administered. Recommendation Rating C.

### Is a low-iodine diet necessary before remnant ablation?

Contamination with stable iodine might theoretically influence the uptake of diagnostic or therapeutic doses of RAI [50–52]. Based on this assumption, several centers advocate preparation of the patients with a low-iodine diet and recommend avoiding iodine contamination (intravenous contrast agents, amiodarone, or other iodine-containing drugs) prior to RAI therapy. However, no prospective study has ever determined the cut off at which interference may actually occur. In a retrospective study, aimed to compare different levels of urinary iodine excretion on the results of thyroid ablation in patients not prepared with low-iodine diet, the authors found no influence of different levels of urinary iodine on the outcome of thyroid ablation up to urinary iodine levels exceeding 350 µg/day of dietary iodine. In addition, the median level of iodine urinary content in Italy is as low as in mild iodine-deficient countries [53] and thus, there is no reason to limit the diet and the use of iodized salt in our country. In any case, measurement of urinary iodine excretion (when available) before remnant ablation may help in detecting the few cases with significant iodine contamination.

## Recommendation 6

At least in countries with mild or moderate iodine deficiency, there is no need to prescribe a low-iodine diet before remnant ablation. Avoidance of iodine-containing drugs or contrast agents is mandatory. If a suspicion of iodine contamination exists, iodine excretion should be evaluated starting from 20 to 30 days after the withdrawal of the iodine-containing drug or the contrast agent injection. Recommendation Rating: B.

### Post-therapy WBS after remnant ablation

It is recommended to perform a post-therapy WBS within 1 week after RAI therapy. This imaging technique is of paramount importance in confirming the presence and the extent of the thyroid remnant and may disclose the presence of unsuspected metastatic foci in 10–26 % of the cases [55], thus allowing the reclassification of the disease stage [56]. Whenever possible, a SPECT-CT can be useful instead of a planar WBS to better define the neck uptake and distinguish the remnant from local lymph node or paratracheal tumor [57].

## Recommendation 7

A post-therapy WBS is recommended following RAI remnant ablation. This is typically done 3–7 days after the therapeutic dose is administered. Recommendation Rating: A.
